# Socioeconomic inequality of overweight and obesity of the elderly in Iran: Bushehr Elderly Health (BEH) Program

**DOI:** 10.1186/s12889-016-3912-1

**Published:** 2017-01-13

**Authors:** Alireza Raeisi, Mohammadbagher Mehboudi, Hossein Darabi, Iraj Nabipour, Bagher Larijani, Neda Mehrdad, Ramin Heshmat, Gita Shafiee, Farshad Sharifi, Afshin Ostovar

**Affiliations:** 1 The Persian Gulf Tropical Medicine Research Center, The Persian Gulf Biomedical Sciences Research Institute, Bushehr University of Medical Sciences, Po box: 7514763448, Imam Khomaini Street, Bushehr, Iran; 2 Endocrinology & Metabolism Research Center, Endocrinology and Metabolism Clinical Sciences Institute, Tehran University of Medical Sciences, Tehran, Iran; 3 Elderly Health Research Center, Endocrinology and Metabolism Population Sciences Institute, Tehran University of Medical Sciences, Tehran, Iran; 4 Chronic Diseases Research Center, Endocrinology and Metabolism Population Sciences Institute, Tehran University of Medical Sciences, Tehran, Iran

**Keywords:** Obesity, Overweight, Socioeconomic Status, Elderly, Iran

## Abstract

**Background:**

The objective of this population-based, large sample size study was to investigate the socioeconomic inequality of overweight and obesity among the elderly in Iran.

**Methods:**

Baseline data of 3000 persons aged ≥60 years who participated in the Bushehr Elderly Health (BEH) program was analyzed. Overweight and obesity were defined as a body mass index (BMI) equal to or higher than 25 and 30, respectively. Socioeconomic status (SES) was measured by an asset index, constructed using principal component analysis, income, education level, and employment status. The Concentration Index and the Lorenz curve were used to illustrate the levels of inequality for overweight and obesity by gender.

**Results:**

The frequencies among men and women were, respectively, 840 (57.7%) and 1131 (73.2%), *P* < 0.001, for overweight, and 211 (14.7%) and 511 (33.7%), *P* < 0.001, for obesity. There were direct associations between asset index quintiles and both overweight and obesity among both genders (*Ps* for trend <0.01) except for obesity among men (*P* for trend = 0.118). The overall Concentration Indices for overweight and obesity were 0.031 (95%CI = 0.016–0.046, *P* < 0.001) and 0.041 (95%CI = 0.004–0.078, *p* = 0.028), respectively.

**Conclusion:**

Findings support the direct relationship between SES and obesity among women as previously reported in developing countries.

## Background

Nowadays, non-communicable diseases are the main public health concerns. These diseases are the cause of about 5% of deaths in the world [1]. Overweight and obesity are the main risk factors for many non-communicable diseases like diabetes, cardiovascular diseases, musculoskeletal disorders, and some types of cancers [2].

Statistics show, in both developed and developing countries, that the prevalence of overweight and obesity is growing. In addition, from 1980 to 2013, there was an increase in the worldwide prevalence of overweight and obesity—from 28.8 to 36.9% in men and from 29.8 to 38.0% in women [3]. It is estimated that, in 2010, overweight and obesity caused 3.4 million deaths and led to 3.9% of the years of life lost (YLL) and 3.8% of disability adjusted life years (DALYs) in the world [3]. Twenty-three percent of ischemic heart diseases, 44% of diabetes, and 7 to 14% of some types of cancer are attributed to overweight and obesity [4]. Obesity was once associated with high-income countries, but now it is prevalent in low-income countries as well. There are countries in which obesity kills more people than underweight. According to the World Health Organization (WHO), 65% of the world’s population lives in high-income and middle-income countries [4]. Therefore, the distribution of overweight and obesity is not equal in all countries, and there is some kind of inequality in its burden among countries.

Health inequalities refer to differences in health status of certain population groups, particularly between people of different socio-economic groups, that can be avoidable and unfair [5]. Various indicators have been used to determine socioeconomic status (SES) of individuals including income, education, occupation, asset/wealth, race/ethnicity, and housing characteristics. Each indicator stratifies people in a different way that affects health outcomes differently; however, these indicators are highly correlated [6]. For example although income provides a direct measurement of SES, it is advised to use asset because it provides more valid SES indicator in developing countries [7].

The prevalence of obesity varies across societies and even different groups in a society. In developed countries, obesity is considered to affect people of lower SES more [8], but there is still a debate as to whether obesity affects poor or rich people more in developing countries. According to a review study conducted in 1989, there was a positive association between SES and obesity in developing countries. Results of that study showed that obesity was a problem of richer people in the studied countries [9]. Recent evidence also indicates that the burden of obesity in developing countries tend to shift to some specific SES groups [10–12].

On the other hand, people all around the world are living longer. In 2015, there were about 900 million people aged 60 years and above in the world, and it is expected that this number will reach 2 billion people by 2050 [13]. Additionally, there were 125 million people aged 80 years and above in the world, and it is estimated that by 2050 that number will grow to about 434 million people. This process of shifting the distribution of population to older ages—which is known as *population aging*—started in high-income countries; now, middle-income and low-income countries are also expecting a growing percentage of the elderly in their populations. According to WHO, by the middle of the century, many countries like Chile, China, Iran, and Russia will have a proportion of elderly people to the general population similar to that in Japan. Population aging can be seen as a success for public health policies and socioeconomic development [13].

Although many studies have investigated the relationship between SES and obesity, there are few reports on that relationship in the elderly. Moreover, some reports from developing countries suffer from methodological limitations in selection of participants or measurement of SES indicators. The objective of this population-based, large sample size study was to investigate the socioeconomic inequality of overweight and obesity among the elderly in Iran, a developing country.

## Methods

Baseline data of the Bushehr Elderly Health (BEH) program was analyzed in this study. The BEH Program is a population-based, prospective cohort study currently being conducted in Bushehr, Iran. The study’s rationale, design, and preliminary results were described elsewhere [14]. A total of 3000 persons aged ≥60 years were selected through a multistage, stratified cluster random sampling method from an estimated population of about 10 000 individuals. The number of participants was proportional to the number of households residing in each of 75 strata of Bushehr port, Iran.

Overweight and obesity were defined as a body mass index (BMI) equal to or higher than 25 and 30, respectively. Height was measured using a stadiometer and weight was measured after removing heavy outer garments and shoes. The BMI was defined as weight (kg) divided by the square of height (m).

We used four widely used SES indicators including: asset index, income level, educational level, and employment status in this study. To collect household asset data, we used a questionnaire in which we asked if participants owned any of 20 household assets (See Table 2 for the list of household assets). We also surveyed participants’ incomes, educational levels, and employment status (See Table 1).

**Table 1:** Baseline socio-demographic characteristics of participants in Bushehr Elderly Health Program

Characteristics [N (%)]	Men [1455 (48.5)]	Women [1545 (51.5)]
Age group		
≤ 64	616 (42.3)	674 (43.6)
65–69	317 (21.8)	378 (24.5)
70–74	230 (15.8)	200 (12.9)
75–79	166 (11.4)	181 (11.7)
≥ 80	126 (8.7)	112 (7.2)
Marital status		
Single	5 (0.3)	20 (1.3)
Married	1378 (94.7)	884 (57.2)
Widowed	68 (4.7)	619 (40.1)
Divorced	4 (0.3)	22 (1.4)
Current occupation		
Employed	133 (9.1)	23 (1.5)
Retired	1195 (82.1)	126 (8.2)
Unemployed^*^	127 (8.7)	1396 (90.4)
Education		
No education	315 (21.6)	777 (50.3)
Primary school	400 (27.5)	459 (29.7)
Secondary School	276 (19.0)	151 (9.8)
High school	287 (19.7)	125 (8.1)
University	177 (12.2)	33 (2.1)
Basic health insurance	1393 (95.7)	1479 (95.7)

^*^homemaker for female

### Statistical analysis

Principal component analysis (PCA) was used to construct an asset index for each individual. We then grouped the participants into quintiles according to their asset indices [15]. PCA has been widely used to construct SES index from binary asset ownership variables such as those collected in this study in developing countries. This method reduces the number of variables into a smaller number of dimensions named principal components (PC) which are uncorrelated to each other using a multivariate statistical technique. A principal component is a linear weighted combination of household asset variables [16]. A total of 6 PCs were retained with higher eigenvalues (>1) accounting for 52.0% of the variance.

The prevalence of overweight and obesity was defined as the number of participants with overweight or obesity divided by total participants, grouped by sex, age range, and socioeconomic quintiles. Pearson’s correlation coefficient was used to assess the association of BMI and asset index. A test for trends across ordered groups was used to assess if there was a linear trend for overweight or obesity prevalence across the socioeconomic levels. Additionally, Pearson’s chi-squared test was used to compare the prevalence between employment status groups.

The Concentration Index, as a summary measure of health inequality, was calculated in participants with overweight and obesity by using the convenient regression method. The asset index was calculated using PCA and was considered to be the socioeconomic index in this analysis. The Lorenz curve was used to graphically present the inequality as a plot of (1) cumulative proportion of the population ranked by asset index against (2) cumulative proportion of overweight or obesity in the population. The Concentration Index is defined as twice the area between the Lorenz curve and the line of equality. The Concentration Index takes a negative sign when the Lorenz curve is above the line of equality indicating the concentration of health variable (obesity in this study) concentrates among low socioeconomic groups and vice versa [17].

*P*-values <0.05 were considered statistically significant, and statistical analyses were performed using the Stata statistical package, release 13.

## Results

A total of 1455 (48.5%) men and 1545 (51.5%) women with mean age and standard deviation (SD) of 67.9 ± 7.1 participated in this study. Other baseline and socio-demographic characteristics of the participants are shown in Table 1.

Table 2 presents the list of home assets identified by participants and used to construct the asset index, including the number of individuals who had each item.

**Table 2:** Frequency of Home Assets owned by participants

Asset	Frequency^*^	Asset	Frequency^*^
Refrigerator	2998 (99.93)	PC/Laptop	1075 (35.83)
Freezer	2903 (96.77	Internet/Wi Fi	1045 (34.83)
Black/White TV	23 (0.77)	Radio	1330 (44.33)
Color TV	2034 (67.80)	Vacuum	2593 (86.43)
LCD TV	1246 (41.53)	Mobile phone	2850 (95.00)
Landline	2670 (89)	Bicycle	170 (5.76)
Washing machine	2655 (88.5)	Motorcycle	516 (17.20)
Dish washing machine	203 (6.77)	Car	1339 (44.63)
Sofa	1458 (48.60)	Boat	27 (0.90)
Microwave	783 (26.10)	Watch	1591 (53.03)

^*^[N (%)]

**Fig. 1:**
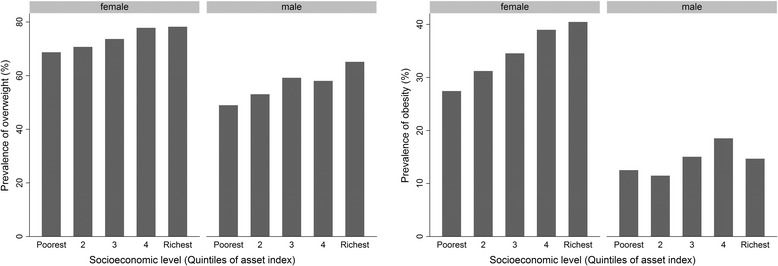
Prevalence of overweight (*left*) and Obesity (*right*) in different levels of socio-economic status by sex

The mean and SD of BMI was 25.9 ± 4.1 kg/m^2^ for men and 28.3 ± 5.3 kg/m^2^ for women. The frequency of overweight and obesity were 840 (57.7%) and 211 (14.7%) among men, and 1131 (73.2%) and 511 (33.7%) among women, respectively. Figure 1 illustrates the prevalence of overweight and obesity over asset index quintiles by sex groups, and Table 3 shows the prevalence of overweight and obesity at different socioeconomic levels.

**Table 3:** Sex-specific Prevalence of Overweight and Obesity by Different Socio-economic Status Indices

		Overweight N (%)				Obesity N (%)			
		Men	*P*	Women	*P*	Men	*P*	Women	*P*
Asset Index	Quintile 1	116 (49.0)	<0.001^*^	283 (68.7)	0.001^*^	29 (12.5)	0.118^*^	110 (27.4)	<0.001^*^
	Quintile 2	131 (53.0)		215 (70.7)		28 (11.5)		93 (31.2)	
	Quintile 3	171 (59.2)		229 (73.6)		43 (15.0)		106 (34.5)	
	Quintile 4	181 (58.0)		228 (77.8)		57 (18.5)		113 (39.0)	
	Quintile 5	241 (65.1)		176 (78.2)		54 (14.7)		89 (40.5)	
C Monthly Household Income (10,000 RLS)	<250	68 (53.1)	0.001^*^	171 (65.0)	0.006^*^	14 (11.2)	0.352^*^	63 (25.2)	0.100^*^
	250–500	70 (59.3)		141 (72.7)		17 (14.5)		52 (27.5)	
	500–1000	430 (53.4)		649 (74.5)		113 (14.2)		317 (36.8)	
	1000–2000	243 (66.8)		140 (80.0)		59 (16.3)		68 (39.1)	
	>2000	21 (70.0)		13 (81.3)		6 (20.7)		6 (37.5)	
Education level	No Education	174 (55.2)	0.664^*^	536 (69.0)	0.256^*^	47 (15.3)	0.092^*^	219 (29.1)	0.018^*^
	Primary	227 (56.8)		350 (76.3)		54 (13.6)		161 (35.5)	
	Secondary	159 (57.6)		114 (75.5)		43 (15.8)		63 (42.0)	
	High School	171 (59.6)		104 (83.2)		49 (17.1)		52 (41.6)	
	University	109 (61.6)		27 (81.8)		18 (10.2)		16 (48.5)	
Employment Status	Employed	75 (56.4)	0.762^†^	16 (69.6)	0.452^†^	22 (16.5)	0.173^†^	10 (43.5)	0.122^†^
	Unemployed^**^	70 (55.1)		1017 (72.9)		11 (9.1)		450 (32.9)	
	Retired	695 (58.2)		98 (77.8)		178 (15.0)		51 (40.8)	

^*^Ps = Trend test across ordered groups

^**^Homemaker for women

^†^ P for Pearson Chi-squared test

**Fig. 2:**
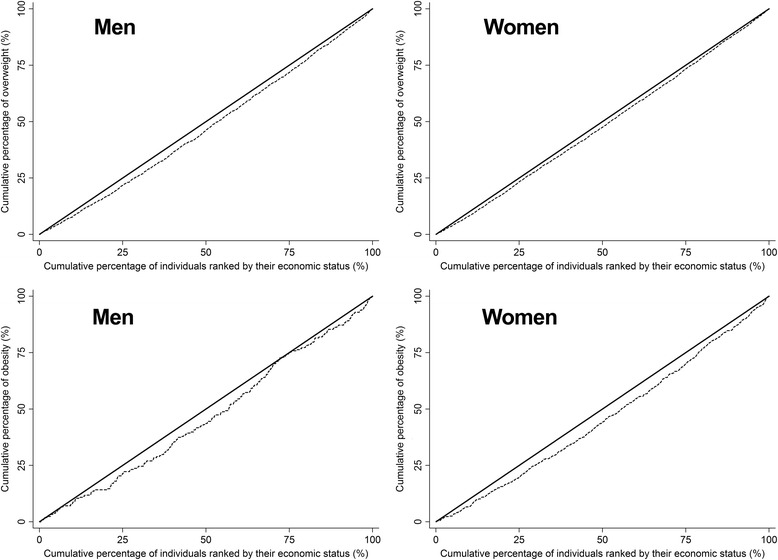
Lorenz curves for overweight (*top*) and obesity (*bottom*) by sex

The overall Concentration Index for overweight was 0.031 (95%CI = 0.016–0.046), *P* < 0.001 [0.056 (95%CI = 0.030–0.081, *P* < 0.001) for men and 0.035 (95%CI = 0.017–0.052, *P* < 0.001) for women]. Furthermore, the overall Concentration Index for obesity was 0.041 (95%CI = 0.004–0.078, *p* = 0.028) [0.071 (95%CI = −0.002–0.143, *p* = 0.055) for men and 0.086 (95%CI = 0.045–0.127, *p* < 0.001) for women]. Figure 2 illustrates the Lorenz curves for overweight and obesity.

## Discussion

Findings of the present study show that the prevalence of overweight and obesity in the elderly was higher in higher socioeconomic groups. The association between SES and overweight and obesity was more obvious in women than in men. Although there were differences in the findings for various SES indicators, the highest degree of inequality was seen among women.

Sobat et al. [9] reported in their literature review that there was a reverse association between SES and obesity among women in developed countries. The association was consistent for men and children. However, the direction of the association was positive in developing countries. Since that time, many studies have investigated the relationship and approved the findings [18, 19]. Findings of the present study are consistent with the previous reports from developing countries; however, the direct association was not seen among men.

Philipson et al. [20] reasoned in their reverse hypothesis that energy imbalance (through increase in intake and decline in consumption) can explain the higher obesity prevalence in higher SES groups in developing countries. Although this is a good explanation for findings among women, it is not well understood why the results are different for men. In the present study, the relationship between SES and obesity prevalence among men resembles the one in previous studies conducted in developed countries. Dinsa et al. [21] also showed that even in the developing world, the relationship between SES and obesity in middle-income countries was mainly mixed among men, compared to the direct relationship among men in low-income countries. The modifying effects of smoking and work-related physical activity on the relationship between SES and obesity have been mentioned; however, none would explain the relationship adequately as to men [21]. Further investigation is needed to explore the mechanisms behind the effects of socioeconomic development on that relationship.

Moreover, findings of the present study show that the relationship between SES and obesity in the elderly resembles that in adulthood. Previous studies in children also showed that, in developed countries, obesity was more prevalent in lower-income people, but in developing countries it was more common in higher-income people [22]. On the other hand, as SES changes during life, risk of obesity changes accordingly [23]. These findings show that the association of SES and obesity seen in adulthood is generalizable to other age groups, such as children and the elderly, just as we found with the elderly in the present study.

Another point in the relationship between SES and obesity is that the direction of relationship depends on the type of SES indicator. Dinsa et al. [21] found that income and wealth were directly associated with obesity in developing countries; however, the association between education and obesity was inverse or non-significant. This finding was consistent among men and women [21]. In the present study, we found that there was a direct, statistically significant association between obesity and assets and also obesity and education among women. The association for income and employment was not statistically significant. There was not, however, any significant association between SES indicators and obesity among men. It seems that there are many complexities in the relationship between SES and obesity that makes that dynamic so difficult to interpret. For example: Aitsi-Selmi et al. [24] found that education modifies the association of wealth and obesity in middle-income countries [25]. Understanding obesogenic mechanisms of SES is necessary in order to interpret the association. It might be necessary to put more emphasis on the social aspects of obesity that cause the attitudes of educated women in developing countries to resemble those of women in developed countries, under the influence of globalization [9, 19]. On the other hand, as societies develop, technological changes push the relationship between SES and obesity from a direct association toward a reverse relationship [19, 20]. However, these changes occur differently according to time, level of development, and other factors such as sex and age. The intermediate pattern observed among men might be a reflection of this changing, dynamic, complex phenomenon.

Exploring the relationship between SES and overweight, as well as obesity, might help us to better understand the mechanism behind this relationship and the resulting inequalities. The findings of the present study showed that there was a direct association between SES and overweight among both men and women. It might be said that as societies develop, the pattern of the relationship between SES and obesity changes more quickly than that between SES and overweight. In other words, overweight is more resistant to the modifying factors. Therefore, any change in the direction of overweight’s relationship with SES would occur at later stages of development. However, few studies have investigated the relationship between SES and overweight.

Finally, the inequality of both overweight and obesity was concentrated in higher SES groups. This is consistent with findings of Alaba and Chola’s study [26] in South Africa that reported a positive, relatively small (particularly for women) Concentration Index for obesity. The Concentration Index observed in the present study was relatively small as well. Concentration Indices for obesity and overweight reported from other studies, mostly from developed countries, were not so large; however, they were mostly negative [27–32].

### Strength and limitations

This is the largest population-based study which has investigated the health of the elderly in Iran. Moreover, measurement of various indicators including assets, income, education, and employment enabled us to more reliably assess socioeconomic level of participants and compare the results. Exact measurement of weight and height in a randomly selected sample with a high response rate (>90%) enabled us to estimate the prevalence of obesity and overweight in various SES groups by age group and gender.

However, as health status in the elderly is highly affected by SES during early life and adulthood, it would be highly informative if we had that SES information. Moreover, because there are recall problems among the elderly, and literacy rates were relatively low, especially among women, there might be some limitations on the validity of answers to some questions (e.g., those for income level, education, and household assets). Both limitations can cause non-differential misclassification of participants into SES groups and introduce an information bias towards the null. However, a very high correlation between various SES variables would guarantee an acceptable reliability.

## Conclusion

Results of the present study support the direct relationship between SES and obesity previously reported in developing countries. However, this study was conducted on elderly people, while many others had been conducted on adults and children. Developing countries, like Iran, should note that as they develop, the concentration of inequality in obesity will move to the poorer people who are the most vulnerable group in the society. Socioeconomic interventions seem necessary to mitigate that inequality and its potential adverse effects.

## Abbreviations

BEH: Bushehr Elderly Health
BMI: Body Mass Index
DALLY: Disability Adjusted Life Years
PCA: Principal Component Analysis
SD: Standard Deviation
SES: Socioeconomic Status
WHO: World Health Organization
YLL: Years of Life Lost
